# Engineering *Escherichia coli* for diagnosis and management of hyperuricemia

**DOI:** 10.3389/fbioe.2023.1191162

**Published:** 2023-05-23

**Authors:** Gozde Gencer, Christopher Mancuso, Koon Jiew Chua, Hua Ling, Cait M. Costello, Matthew Wook Chang, John C. March

**Affiliations:** ^1^ Biological and Environmental Engineering Department, Cornell University, Ithaca, NY, United States; ^2^ Biomedical Engineering Department, Boston University, Boston, MA, United States; ^3^ Synthetic Biology Translational Research Program and Department of Biochemistry, Yong Loo Lin School of Medicine and NUS Synthetic Biology for Clinical and Technological Innovation (SynCTI), National University of Singapore, Singapore, Singapore

**Keywords:** gout, urate oxidase, probiotic, *Bacillus*, pump, sink accumulation

## Abstract

Uric acid disequilibrium is implicated in chronic hyperuricemia-related diseases. Long-term monitoring and lowering of serum uric acid levels may be crucial for diagnosis and effective management of these conditions. However, current strategies are not sufficient for accurate diagnosis and successful long-term management of hyperuricemia. Moreover, drug-based therapeutics can cause side effects in patients. The intestinal tract plays an important role in maintaining healthy serum acid levels. Hence, we investigated the engineered human commensal *Escherichia coli* as a novel method for diagnosis and long-term management of hyperuricemia. To monitor changes in uric acid concentration in the intestinal lumen, we developed a bioreporter using the uric acid responsive synthetic promoter, *pucpro*, and uric acid binding *Bacillus subtilis* PucR protein. Results demonstrated that the bioreporter module in commensal *E. coli* can detect changes in uric acid concentration in a dose-dependent manner. To eliminate the excess uric acid, we designed a uric acid degradation module, which overexpresses an *E. coli* uric acid transporter and a *B. subtilis* urate oxidase. Strains engineered with this module degraded all the uric acid (250 µM) found in the environment within 24 h, which is significantly lower (*p* < 0.001) compared to wild type *E. coli*. Finally, we designed an *in vitro* model using human intestinal cell line, Caco-2, which provided a versatile tool to study the uric acid transport and degradation in an environment mimicking the human intestinal tract. Results showed that engineered commensal *E. coli* reduced (*p* < 0.01) the apical uric acid concentration by 40.35% compared to wild type *E. coli*. This study shows that reprogramming *E. coli* holds promise as a valid alternative synthetic biology therapy to monitor and maintain healthy serum uric acid levels.

## Introduction

Uric acid is formed in the liver, intestines, muscles, and kidneys as a product of purine catabolism. This product remains in the body until approximately two-thirds of it is excreted via the kidneys, and the remaining one third via the intestinal tract (Sorensen, 1965). Most mammals can oxidize uric acid into allantoin via urate oxidase. Allantoin is highly soluble in water and can readily be excreted from the kidneys. Humans do not produce urate oxidase (Maiuolo et al., 2016), a condition which can lead to overproduction or inefficient excretion of uric acid, leading to high concentrations of this compound in blood serum, a condition known as *hyperuricemia*. Hyperuricemia is defined by serum uric acid levels exceeding 360 µM for women and 420 µM for men (Kutzing and Firestein, 2007). Despite its powerful antioxidant properties that counteract the effects of oxidative damage due to atherosclerosis and aging, chronic high concentrations of uric acid have adverse effects on human health (Kutzing and Firestein, 2007). The prevalence of this disease is substantial among adults in the United States, affecting approximately 8.3 million individuals (Zhu et al., 2012). Hyperuricemia is often associated with diseases, such as gout, metabolic syndrome, hypertension, tumor lysis syndrome, chronic kidney, and cardiovascular diseases (Gliozzi et al., 2016). Evidence suggests that elevated uric acid levels in serum can be an independent and significant risk factor for these pathologies (Kang et al., 2002; Bos et al., 2006; Perlstein et al., 2006; Kutzing and Firestein, 2007; Johnson et al., 2013). Lowering uric acid levels has been shown to benefit patients with gout, chronic kidney, and cardiovascular disease (Wortmann, 2002; Goicoechea et al., 2010). Thus, long-term monitoring and lowering of elevated serum uric acid levels may be crucial for diagnosis and effective management of chronic hyperuricemia-related diseases. ‬‬‬‬‬‬‬‬‬‬‬‬‬‬‬‬‬‬‬‬‬‬‬‬‬‬‬‬‬‬‬‬‬‬‬‬‬‬‬‬‬‬‬‬‬‬‬‬

Commonly used hyperuricemia therapeutics aim to lower serum uric acid levels either by *(i)* competing with xanthine oxidase enzymes to reduce the production of this compound in liver (e.g., allopurinol, febuxostat), *(ii)* blocking uric acid transporters (e.g., Human Urate Transporter 1 (URAT1), Glucose Transporter 9 (GLUT9)) to prevent its reabsorption from kidneys (e.g., probenecid, benzbromarone), or *(iii)* metabolizing uric acid via recombinant urate oxidase activity [e.g., pegloticase (Sundy et al., 2007), rasburicase (Ueng, 2005)]. However, such traditional therapies may lead to significant side effects in patients, including hypersensitivity, intolerance, abnormalities in liver function, and immune response (Yang et al., 2012; Gliozzi et al., 2016). For example, allopurinol can cause dose-dependent side effects, especially among patients with renal insufficiency (Hande et al., 1984; Stamp et al., 2011). Alternative therapies for patients intolerant to allopurinol include febuxostat and benzbromarone, but these drugs can lead to abnormalities in renal function tests and even liver toxicity in some patients (Suresh and Das, 2012). For these patients, recombinant urate oxidase therapy provides an alternative. Nevertheless, these drugs proved to be suitable for short-term treatment only (Sundy et al., 2007; Yang et al., 2012). All in all, there is need for additional therapies to provide long-term uric acid homeostasis without these complications.

An alternative target organ for novel diagnosis and long-term management of hyperuricemia is the intestinal tract. There are two major pathways for uric acid removal in the intestine, *(i)* polarized efflux from the basolateral to the apical (luminal) side via transporters like Breast Cancer Resistance Protein BCRP [ATP-binding cassette super-family G member 2 (ABCG2)] (Hosomi et al., 2012) and *(ii)* increased diffusion into intestinal lumen via pH-trapping in the upper intestinal tract (Yun et al., 2017). Clinical trials with a uric acid absorbing hydrogel, known as sevelamer, (Garg et al., 2005; Ohno et al., 2009), and recent research on animal models (Szczurek et al., 2017; Yun et al., 2017), which applied oral recombinant urate oxidase therapy showed that uric acid secretion into the intestinal tract can be enhanced, likely due to the concentration gradient facilitated by luminal uric acid removal. These studies demonstrated a significant decrease in serum uric acid levels following its removal from the intestinal tract. However, only 23% of the subjects, who were treated with sevelamer in clinical trials, experienced a significant reduction (Garg et al., 2005). As for the oral recombinant urate oxidase therapy (Szczurek et al., 2017; Pierzynowska et al., 2020), the presence of proteases in the gastrointestinal tract and the immune response against recombinant proteins are the main obstacles for long-term use of this method (Yang et al., 2012). These emerging treatments present potential solutions for hyperuricemia, but alternative therapies are needed to overcome the body’s own defenses and achieve a clinically significant reduction in serum uric acid levels in majority of the patients.

Next-generation microbiome-based therapeutics aim to take advantage of the close interaction between human health and gut microbiota. One possible approach involves modulating the composition or activity of the microbiota via genetically engineered microbes (Mimee et al., 2016). Engineered microbes can provide non-invasive, cost-effective, and on-site diagnosis and treatment for various human diseases (Archer et al., 2012; Kotula et al., 2014; Duan et al., 2015; Hwang et al., 2017). This study presents the engineered human commensal *E. coli* (i.e., *E. coli* K-12 MG1655, *E. coli* Nissle 1917) as a novel method for non-invasive monitoring of uric acid levels and long-term management of hyperuricemia through the intestinal tract. We reprogrammed *E. coli* to *(i)* monitor changes in uric acid concentration using a bioreporter module, and *(ii)* degrade excess uric acid found in the environment. Moreover, we designed and built an *in vitro* model with human intestinal cell line, Caco-2, to study the transport and degradation of uric acid in an environment mimicking the human intestinal tract. In summary, harnessing genetically engineered *E. coli* for long-term monitoring and degradation of uric acid provides an essential contribution for *in situ* detection and elimination of hyperuricemia-causing disease biomarker through the intestinal tract.

## Results

### Estimation of intestinal uric acid concentration

An estimate of uric acid concentration in the human intestinal lumen is required to develop a bioreporter, which can differentiate between healthy and disease states. Studies (Nishida, 1992; Lin et al., 2002; Perez-Ruiz et al., 2002; Yu et al., 2004) reported different ranges for the daily amount of uric acid removed via renal pathway. In particular, the average urinary uric acid excretion is considered to be in between 600 to 650 mg/d (Nishida, 1992; Lin et al., 2002; Perez-Ruiz et al., 2002; Yu et al., 2004) for healthy individuals and over 1,000 mg/d^32^ for hyperuricemia patients. Using these urinary uric acid excretion values, we calculated the amount of total endogenous uric acid that the human body excretes. As 30% of that total amount is known to be excreted via intestines (Sorensen, 1965), we estimated the intestinal secretion rates of healthy individuals and hyperuricemia patients as 0.1786 mg/min and 0.2976 mg/min, respectively (Supplementary Figure S1). For our calculations, we assumed that the renal to intestinal uric acid excretion ratio is the same for healthy and hyperuricemia cases. However, the intestinal uric acid secretion rate can be higher for hyperuricemia patients as intestinal secretion compensates for renal underexcretion (Vaziri et al., 1995; Perez-Ruiz et al., 2002).

To calculate steady state intestinal uric acid concentrations, we applied a mass balance on uric acid evolution in the intestine using Equation 1. In this equation, *m*_*U*_ indicates the uric acid mass in the intestine, *V*_*int*_ is the intestinal volume, *[U]* is the uric acid concentration, *s* is the intestinal secretion rate, *q* is the intestinal flow rate, and *k* represents the uric acid degradation rate constant. Degradation rate constant *k* was set equal to three times the intestinal flow rate because within the intestines, 75% of uric acid gets degraded by resident bacteria (Sorensen, 1965).

$$\frac{dm_{U}}{dt}=\frac{d\left(V_{\mathrm{int}}\left[U\right]\right)}{dt}=0=s-q\left[U\right]-k=s-4q\left[U\right]$$ (1)

Using long-term intestinal flow rate, 5 mL/min (Fine et al., 1995), we calculated steady state uric acid concentrations as 53.15 μM for healthy individuals and ≥88.57 μM for hyperuricemia patients. These representative intestinal uric acid concentrations enabled us to set a threshold for healthy and disease states.

### Engineering *E. coli* as uric acid bioreporter

**FIGURE 1:**
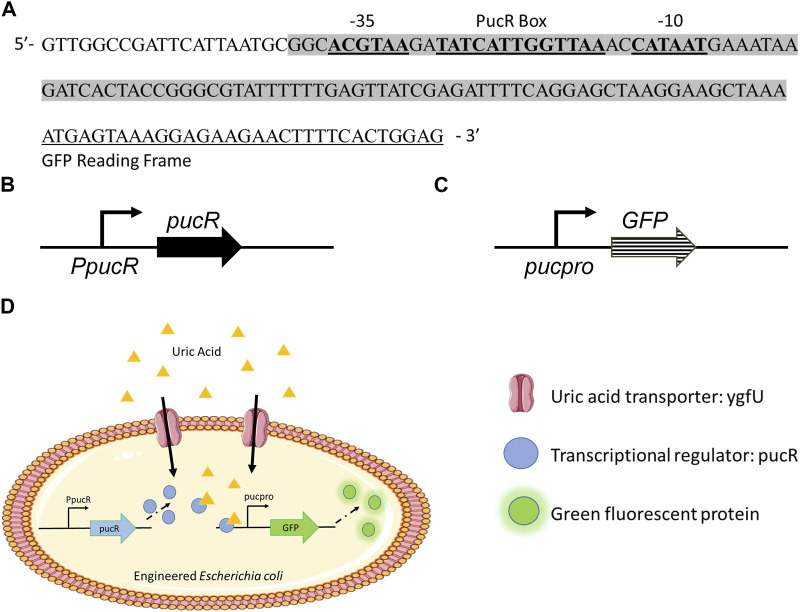
**(A)** Synthetic uric acid responsive promoter, pucpro, sequence. **(B)** pPUCR: pucR expression vector built on pACYC184 backbone. **(C)** pucGFP: pucpro controlled GFP expression vector built on pGFPuv backbone. **(D)** Uric acid detection in engineered *E. coli* using pPUCR and pucGFP constructs.

To develop *E. coli* as a uric acid bioreporter, we designed a synthetic uric acid responsive promoter (*pucpro*) sequence. The rationale behind the design of this promoter was to adapt *Bacillus subtilis*’s purine catabolism regulation into *E. coli* for uric acid detection. Gram positive soil bacterium, *B. subtilis*, utilizes purine bases as a nitrogen source and regulates genes needed for purine catabolism via the PucR protein, the transcriptional activator involved in the induction of the purine degradation pathway. Two factors are important for PucR mediated transcriptional regulation: *(i)* the position of the PucR binding site on the genome relative to transcription start point, and *(ii)* the presence of purine degradation products, such as uric acid, acting as an effector molecule for PucR activation (Beier et al., 2002). To build the *pucpro* promoter, we replaced the spacer between −35 and −10 consensus sequences of constitutive chloramphenicol (CAT) promoter with pucR box from *B. subtilis* 168 (Figure 1A). Our bioreporter module consists of two constructs, *(i)* pPUCR (Figure 1B) and *(ii)* pucGFP (Figure 1C), which enable PucR protein production and uric acid induced green fluorescent protein (GFP) expression, respectively. The uric acid detection mechanism of this bioreporter module can be seen in Figure 1D. Uric acid in the environment is transported into the cell via native *E. coli* uric acid transporter, YgfU. Once inside the cell it binds to the transcriptional regulator protein PucR. This complex, which is now active, induces transcription of GFP by binding to the pucR box on synthetic *pucpro* promoter. Finally, the amount of GFP inside the cell can be measured to evaluate the performance of uric acid bioreporter.

**FIGURE 2:**
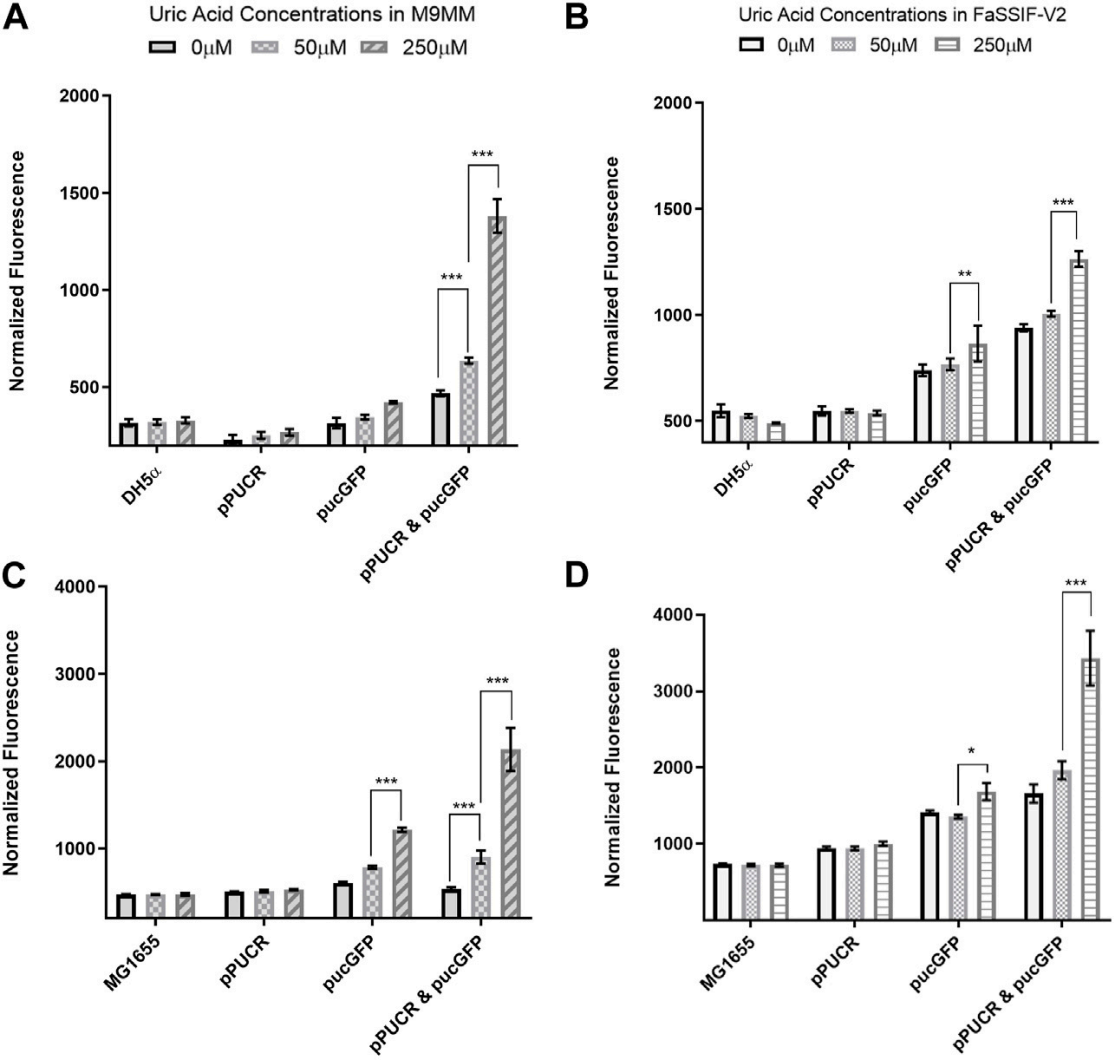
GFP assay results in M9MM **(A,C)** and FaSSIF-V2 **(B,D)** media. *E. coli* DH5a **(A,B)** and *E. coli* K-12 MG1655 **(C,D)** bioreporter strains’ (pPUCR & pucGFP) response to changes in uric acid concentration is compared to control groups, which are (i) native *E. coli* without plasmids, (ii) *E. coli* with pPUCR vector only, and (iii) *E. coli* with pucGFP vector only. Relative fluorescence of each sample is normalized to cell density, OD600. Each bar shows the mean normalized fluorescence and s.d. (error bars) obtained from 4 biological replicates. * indicates *p* < 0.05, ** indicates *p* < 0.01, and *** indicates statistical significance at *p* < 0.001. Statistical analysis: one-way ANOVA is combined with the Bonferroni multiple comparisons test (a = 0.05).

**FIGURE 3:**
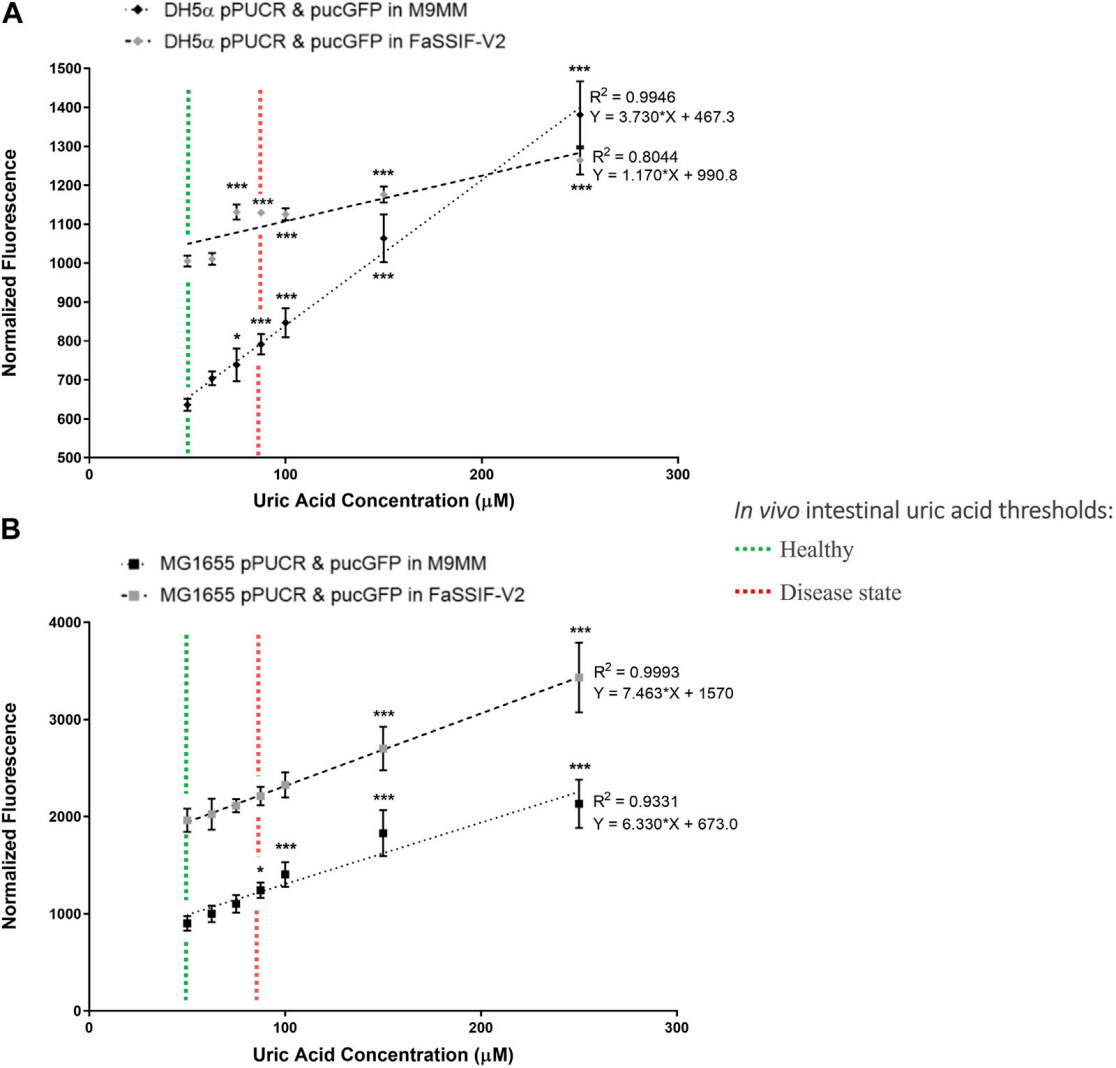
*E. coli* DH5a **(A)** and *E. coli* K-12 MG1655 **(B)** uric acid bioreporter characterization in M9MM and FaSSIF-V2 in response to increasing uric acid concentrations: 50p.M, 62.5p.M, 75p.M, 87.5p.M, 100p.M, 150p.M, and 250p.M. Each data point shows the mean normalized fluorescence and s.d. (error bars) obtained from 4 biological replicates. The mean of each data point was compared to the mean of the control group, 50p.M uric acid. * and *** indicates statistical significance at *p* < 0.05 and *p* < 0.001 respectively. Statistical analysis: one-way ANOVA is combined with the Bonferroni multiple comparisons test (a = 0.05).

**FIGURE 4:**
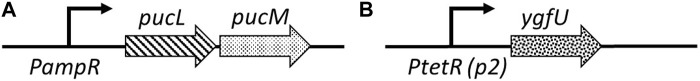
**(A)** pBR-pucLM: pucL-pucM expression vector built on pBR322 backbone. pucLM gene fusion amplified from *B. subtilis* 168 genome. **(B)** pAC-ygfU: ygfU expression vector built on pACYC184backbone. ygfU gene amplified from *E. coli* K-12 MG1655 genome.

**FIGURE 5:**
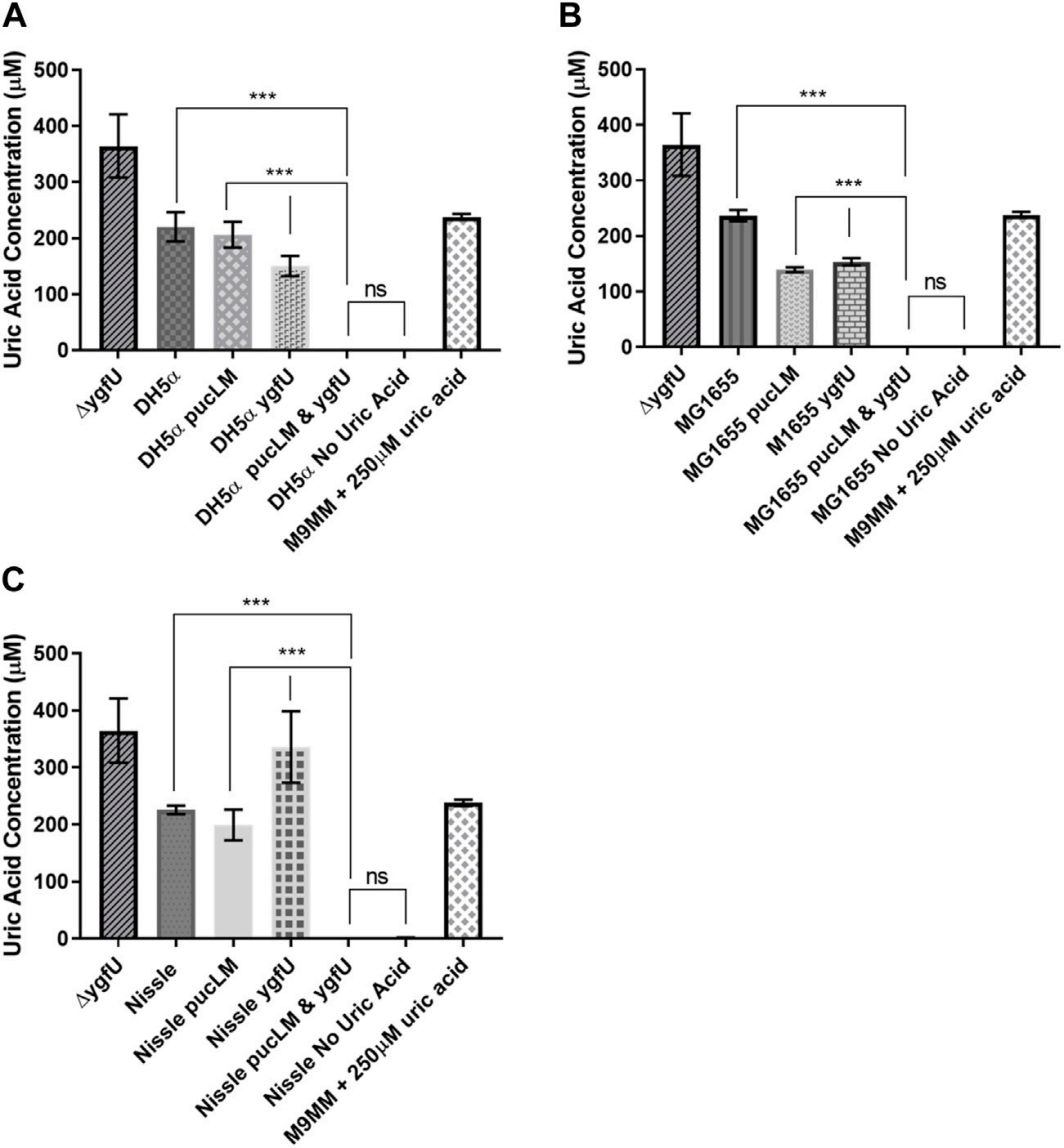
Colorimetric uric acid assay results for *E. coli* DH5a **(A)**, *E. coli* K-12 MG1655 **(B)**, and engineered *E. coli* Nissle 1917 **(C)** reprogrammed with uric acid degradation module components. Each sample was incubated in 250ILIM uric acid supplemented M9MM for 24 h. Each bar shows the mean uric acid concentration and s.d. (error bars) obtained from 2 biological replicates. *** indicates statistical significance at *p* < 0.001 and ns indicates no statistical difference between the samples. Statistical analysis: one-way ANOVA is combined with the Bonferroni multiple comparisons test (a = 0.05).

**FIGURE 6:**
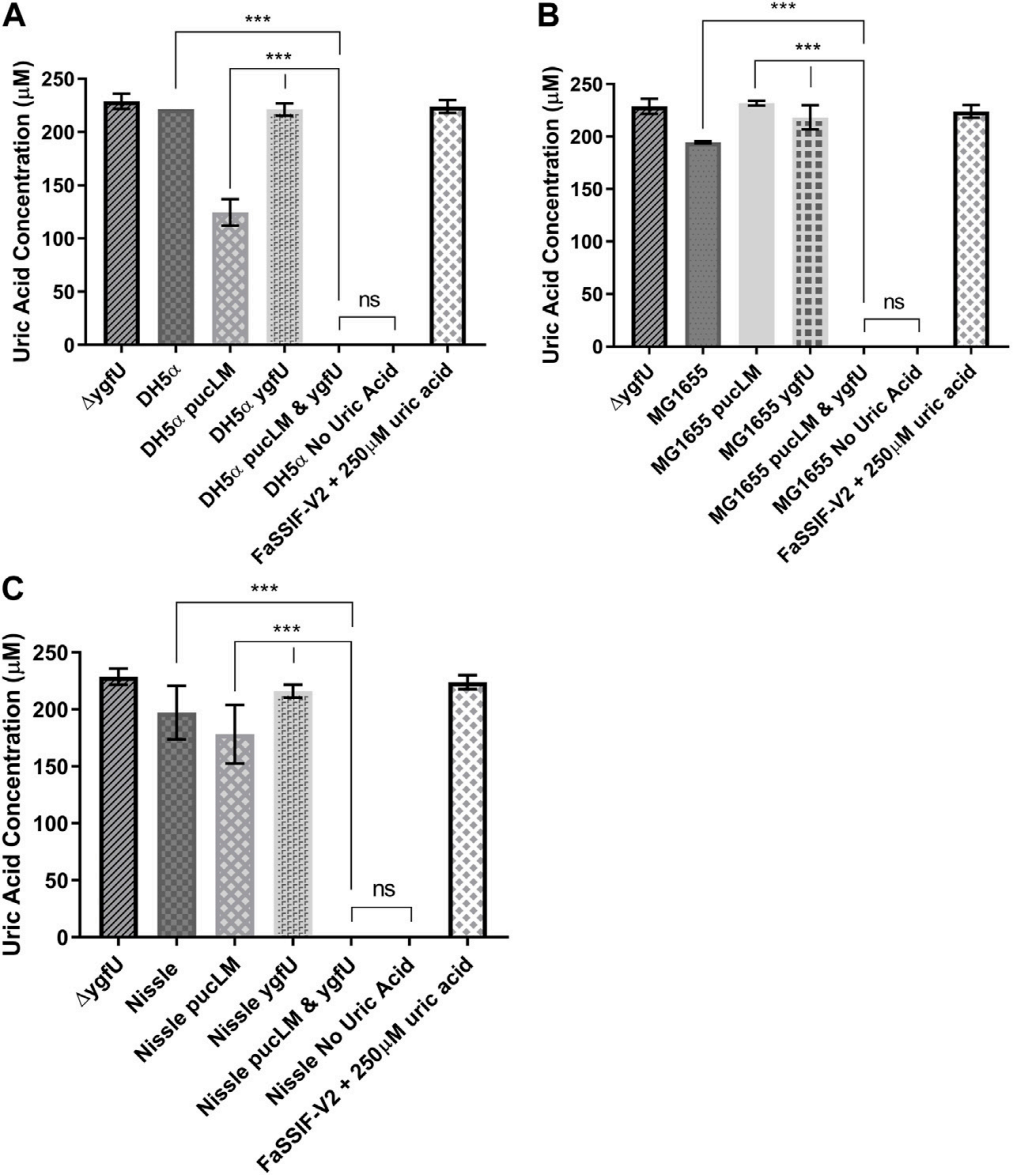
Colorimetric uric acid assay results for *E. coli* DH5ct **(A)**, *E. coli* K-12 MG1655 **(B)**, and engineered *E. coli* Nissle 1917 **(C)** reprogrammed with uric acid degradation module components. Each sample was incubated in 25011M uric acid supplemented FaSSIF-V2 for 24 h. Each bar shows the mean uric acid concentration and s.d. (error bars) obtained from 2 biological replicates. *** indicates statistical significance at *p* < 0.001 and ns indicates no statistical difference between the samples. Statistical analysis: one-way ANOVA is combined with the Bonferroni multiple comparisons test (a = 0.05).

**FIGURE 7:**
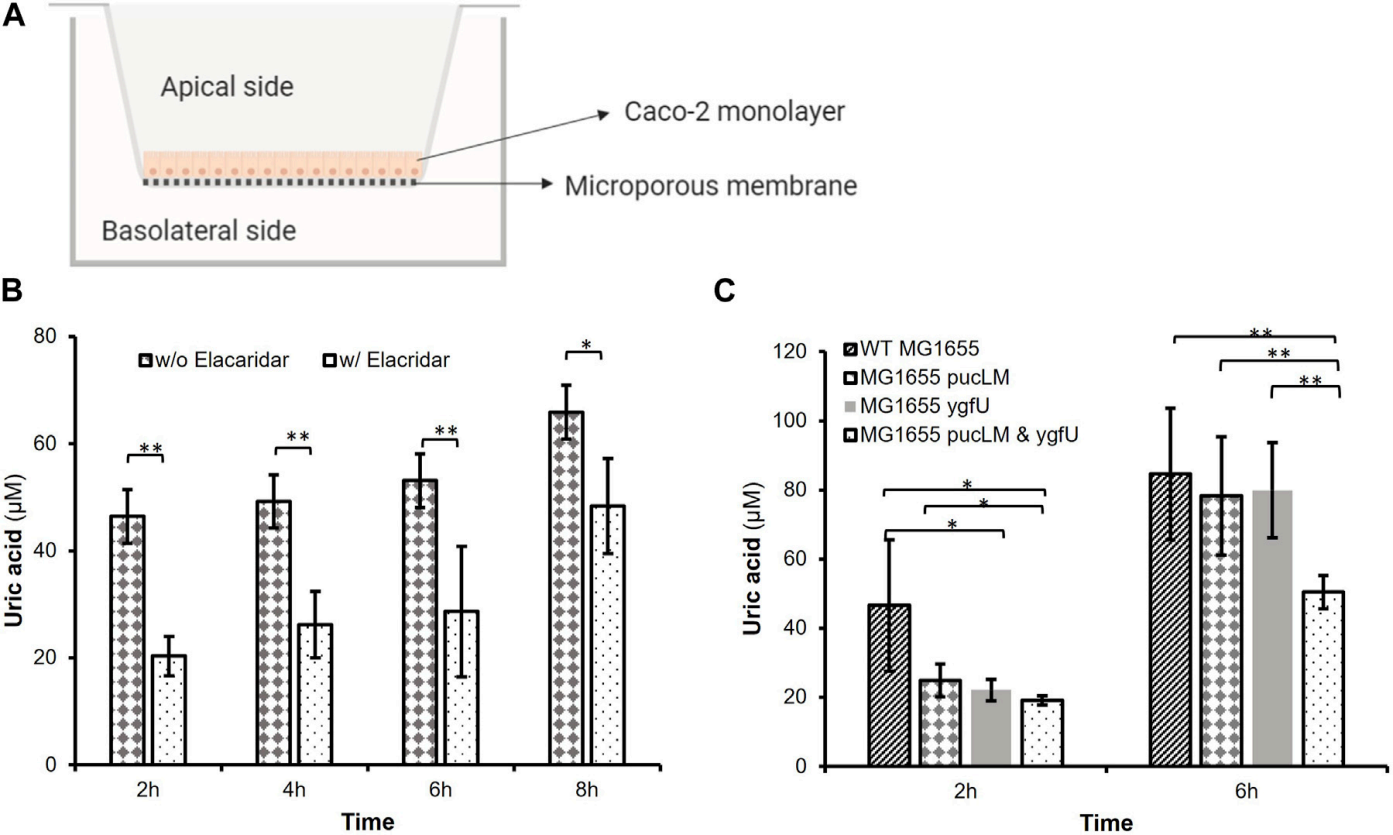
**(A)** *In vitro* Caco-2 model for uric acid transport and degradation experiments. **(B)** Uric acid transport through Caco-2 monolayer over time. Supplementing the basolateral compartment with 2.5 mM uric acid achieved uric acid levels greater than 46.44 iuM on the apical side. Elacridar supplemented samples transported significantly less uric acid from basolateral to apical side. **(C)** Remaining apical uric acid in the presence of bacteria. Engineered *E. coli* K-12 MG1655 (MG1655 pucLM & ygfU) had 40.35% less uric acid compared to wild-type MG1655 after 6 h. Each bar shows the mean uric acid concentration and s.d. (error bars) obtained from 6 biological replicates. * and ** indicates statistical significance at *p* < 0.05 and *p* < 0.01 respectively. Statistical analysis: unpaired Student’s t-test (a = 0.05).

Laboratory strain *E. coli* DH5α and human commensal strain *E. coli* K-12 MG1655 were used as hosts for our uric acid bioreporter module. To quantify the bioreporter’s performance in these bacteria, relative GFP fluorescence of each sample was measured and normalized to cell density. In the first GFP assay (Figure 2), the bioreporter and control strains response to changes in uric acid concentration (0 μM, 50 μM, and 250 μM) was compared under two conditions: *(i)* M9 minimal media (M9MM) to ensure that uric acid is the effector molecule, and *(ii)* fasted state simulated intestinal fluid (FaSSIF-V2) to mimic human intestinal fluid conditions *in vitro*. Both DH5α (Figures 2A, B) and K-12 MG1655 (Figures 2C, D) bioreporter strains produced significantly more GFP protein (*p* < 0.001) when uric acid in the environment increased from 50 μM to 250 μM. The MG1655 strain exhibited higher overall expression of GFP when compared to DH5α (Figure 2). The control group carrying the pucGFP vector had the same type of increase in GFP production (Figures 2B–D), but this response was significantly less (*p* < 0.001) compared to combined effect of pPUCR and pucGFP in the cell. The response observed with pucGFP carrying control might be due to presence of native *E. coli* proteins with *pucpro* promoter binding capability. On the other hand, native *E. coli* and *E. coli* carrying the pPUCR vector, did not show any difference in their GFP fluorescence in response to changing uric acid levels.

In the second round of GFP assays (Figure 3), the bioreporter module was characterized in M9MM and FaSSIF-V2 media for its sensitivity to increasing uric acid concentrations (50 μM, 62.5 μM, 75 μM, 87.5 μM, 100 μM, 150 μM, and 250 μM). Linear regression analysis enabled us to calculate the goodness of fit (R^2^), as well as study the relationship between normalized GFP fluorescence and increasing uric acid concentrations. Results showed that the bioreporter module in both bacterial strains can detect changes in uric acid in a dose-dependent manner. Engineered DH5α significantly differentiated (*p* < 0.001) between healthy (∼53 μM) and disease state (≥88 µM) intestinal threshold values both in M9MM and FaSSIF-V2 (Figure 3A). Engineered K-12 MG1655 significantly differentiated (*p* < 0.05) between healthy and disease states in M9MM (Figure 3B). However, compared to DH5α, bioreporter module in K-12 MG1655 was less sensitive to changes in uric acid under simulated intestinal fluid (FaSSIF-V2) conditions. Still in this media, MG1655 managed to significantly differentiate (*p* < 0.001) between low (50 μM) and high levels of uric acid (≥150 μM) (Figure 3B).

Using the same experimental setup and cloning as for MG1655 and DH5α, we tested the probiotic *E. coli* Nissle 1917, however results suggested that Nissle was not suitable to be used as a uric acid bioreporter (Supplementary Figure S2).

### Engineering *E. coli* to lower serum uric acid levels

Clinical trials with a uric acid absorbing hydrogel (Garg et al., 2005; Ohno et al., 2009) and recent studies in animal models (Szczurek et al., 2017; Yun et al., 2017) demonstrated the important role of intestinal uric acid clearance in hyperuricemia prevention. To regulate serum uric acid levels via the intestinal lumen, we reprogrammed commensal *E. coli*, a native component of human gut microbiota, for enhanced uric acid degradation. Our uric acid degradation module consists of two constructs: *(i)* pBR-pucLM (Figure 4A) enables constitutive expression of recombinant *B. subtilis* urate oxidase in *E. coli* for degradation of uric acid into allantoin, and *(ii)* pAC-ygfU (Figure 4B) enables overexpression of *E. coli* K-12 uric acid transporter, YgfU, for increased uptake of uric acid into the cell (Papakostas and Frillingos, 2012).

*E. coli* strains DH5α, MG1655, and the probiotic Nissle 1917 were used as host strains for our uric acid degradation module. These engineered bacteria along with the control groups were tested in 250 μM uric acid supplemented M9MM and FaSSIF-V2 media. To measure the amount of uric acid remaining in M9MM and FaSSIF-V2 after 24 h of incubation, colorimetric uric acid assays were performed on each sample. Uric acid supplemented media with no cells was used as a negative control to ensure that uric acid was stable in these growth conditions until the colorimetric assay was performed. Assay results demonstrated that reprogrammed *E. coli* DH5α (Figure 5A; Figure 6A), *E. coli* K-12 MG1655 (Figure 5B; Figure 6B), and *E. coli* Nissle 1917 (Figure 5C; Figure 6C) degraded all the uric acid found in the environment after 24 h of incubation in M9MM and FaSSIF-V2, which was significantly lower (*p* < 0.001) compared to wild-type *E. coli* strains and negative control groups harboring pBR-pucLM or pAC-ygfU construct only. The same assay detected higher uric acid concentrations for control strain, Δ*ygfU*, compared to wild-type strains. This can be explained with the ongoing uric acid production via purine catabolism inside the cell while the uric acid transport for degradation is blocked due to lack of YgfU transporter.

### Designing an *in vitro* Caco-2 model for uric acid transport and degradation

The efflux transporter BCRP is expressed abundantly at the apical membrane of human small intestinal epithelial cells and contributes to intestinal excretion of uric acid (Hosomi et al., 2012). Studies on animal models (Hosomi et al., 2012) demonstrated that lack of BCRP causes a decrease in intestinal uric acid secretion, and consequently a significant increase in serum uric acid levels. Based on BCRP’s crucial role in intestinal uric acid transport, we designed an *in vitro* model of human intestinal epithelium using the BCRP expressing cell-line, Caco-2, a well-established model of human epithelial cells for transport and permeability studies. This human intestinal epithelial cell-line shows polarized uric acid flux from basolateral to apical membrane (Hosomi et al., 2012), mimicking the excretion of uric acid into human intestinal lumen. Using this model (Figure 7A), we studied uric acid transport and its degradation via our engineered *E. coli* strain K-12 MG1655 in an environment that mimics the human intestinal tract.

We verified abundant expression levels of BCRP in Caco-2 cells via FACS analysis. (Supplementary Figure S3). Next, Caco-2 cells were cultured on Transwell^®^ inserts for uric acid transport and degradation experiments. The basolateral membrane was incubated with uric acid and the apical membrane was incubated with or without a potent BCRP inhibitor, elacridar, to demonstrate that uric acid transport across the monolayer is predominantly due to BCRP activity. Colorimetric uric acid assay results (Figure 7B) demonstrated that supplementing the basolateral compartment with 2.5 mM uric acid achieved uric acid levels greater than 46.44 μM on the apical side (Supplementary Figure S4). Moreover, after 2–6 h incubation with elacridar-supplemented Caco-2 monolayers transported 46.7–56.2% less (*p* < 0.01) uric acid from the basolateral to apical side (Figure 7B). This demonstrated the importance of BCRP-mediated uric acid transport for polarized flux of this molecule.

In the second experimental setup, we investigated the uric acid degradation potential of engineered *E. coli* strain K-12 MG1655, which was carrying pBR-pucLM and pAC-ygfU constructs. Based on the data from uric acid transport experiments, we incubated the Caco-2 monolayer with 2.5 mM uric acid and bacteria for 6 h, then measured uric acid concentrations via colorimetric uric acid assay. Results demonstrated that apical chambers with engineered *E. coli* K-12 MG1655 (MG1655 pucLM & ygfU) had 40.35% less (*p* < 0.01) uric acid compared to wild-type MG1655 after 6 h (Figure 7C).

## Discussion

The prevalence of hyperuricemia has increased over the last few decades (Suresh and Das, 2012). Traditional drugs for long-term management of this disease target uric acid metabolism and excretion. Patients using these conventional treatment methods may suffer from refractory hyperuricemia due to hypersensitivity or intolerance (Gliozzi et al., 2016). Emerging treatments like uric acid absorbing hydrogel (Garg et al., 2005; Ohno et al., 2009) and pegylated recombinant urate oxidase (Sundy et al., 2007) provide alternative therapies for patients that suffer from these side effects. However, the effectiveness of the former approach is limited (Garg et al., 2005) and the latter treatment is not suitable for long-term use, because many patients develop immune response against this recombinant protein (Sundy et al., 2007; Suresh and Das, 2012). Thus, novel therapies are needed to achieve long-term uric acid homeostasis in majority of hyperuricemia patients.

An alternative target organ for reducing serum uric acid levels below the pathological threshold is the intestinal tract. Evidence from multiple studies (Garg et al., 2005; Ohno et al., 2009; Szczurek et al., 2017; Yun et al., 2017) suggest that uric acid secretion into intestinal tract can be enhanced based on the uric acid gradient between the blood circulation and lumen. In this study, we investigated the potential use of engineered human commensal *E. coli* as a novel method to monitor and regulate serum uric acid levels through the human intestinal tract.

To monitor uric acid levels both in hyperuricemia patients and individuals with epidemiological risk factors (e.g., genetic variation in BCRP and SLC2A9, body mass index, alcohol intake (Nakayama et al., 2014; Mandal and Mount, 2015)), we reprogrammed *E. coli* as a uric acid bioreporter. To build this bioreporter module, we constructed a uric acid responsive synthetic promoter, *pucpro*, which controls the expression of reporter protein GFP. The design of *pucpro* promoter was based on the PucR mediated transcriptional regulation of *B. subtilis* purine catabolism genes. Purine degradation products, such as uric acid, act as the effector molecule for PucR-activated transcription (Beier et al., 2002). The position of the PucR binding site (pucR box) relative to consensus promoter elements and the transcriptional start site determines the nature of the regulation. Beier et al. (2002) demonstrated that PucR represses gene expression when the PucR box is found downstream of the transcriptional start site or when it overlaps with the −35 element, blocking the movement of RNA polymerase. On the other hand, PucR binding induces transcription when the pucR box is placed upstream of the −35 promoter element. By placing the pucR box between −10 and −35 consensus sequences on constitutive chloramphenicol promoter, we expected to observe a repression in GFP expression in response to elevated uric acid levels. However, our initial results (Figure 2) demonstrated that PucR protein induces transcription from the *pucpro* promoter in the presence of uric acid. One possible explanation for this type of positive regulation can be conformational changes on DNA, enhancing RNA polymerase machinery’s assembly following PucR binding to *pucpro* (Brown et al., 2003).

Further characterization of bioreporter module (Figure 3) in *E. coli* DH5α and MG1655 indicated that these engineered strains can detect changes in uric acid concentration in a dose-dependent manner. Even though the bioreporter module in MG1655 was less sensitive to changes in uric acid concentration in FaSSIF-V2 media, this strain’s ability to significantly differentiate (*p* < 0.001) between low (50 μM) and high levels of uric acid (≥150 μM) under simulated intestinal conditions is promising for diagnosis of hyperuricemia through the intestinal tract. Multiple studies (Vaziri et al., 1995; Perez-Ruiz et al., 2002) demonstrated that the intestinal uric acid secretion compensates for the insufficient renal elimination. This indicates that disease state uric acid levels may be higher than the threshold (88.7 μM) estimated in this study. Finally, we tested our bioreporter module in probiotic *E. coli* strain Nissle 1917, too, but it did not work as well as it did in MG1655 (Supplementary Figure S2). The reason behind this might be the differences in uric acid production and degradation rates between *E. coli* strains.

To regulate elevated serum uric acid levels through the intestinal tract, we engineered *E. coli* strains DH5α, MG1655, and Nissle 1917 for the enhanced degradation of excess uric acid. Previous studies showed that (Papakostas and Frillingos, 2012) *E. coli* can transport uric acid into the cell via uric acid transporter protein, YgfU (UacT). However, physiological significance of uric acid uptake is yet to be known, because *E. coli* does not encode for a urate oxidase gene for further catabolism of this molecule (Papakostas and Frillingos, 2012). Hennebry et al. (2006) proposed that *E. coli* K-12 utilizes uric acid as an antioxidant and decomposes it into 5-hydroxyisourate (HIU) via oxidation. To enhance uric acid degradation in *E. coli*, we overexpressed uric acid transporter gene *ygfU* and *B. subtilis* 168 urate oxidase genes *pucL* and *pucM*. A potential advantage of uric acid degradation inside a bacterial cell over the current recombinant urate oxidase treatment strategies (e.g., pegloticase) would be the lack of an immune response against the recombinant protein (Yang et al., 2012; Gliozzi et al., 2016). Colorimetric uric acid assay results (Figures 5, 6) suggest that uric acid degradation via recombinant *B. subtilis* urate oxidase, PucLM, should be coupled with an increased uptake of uric acid for enhanced degradation of this molecule. All three of the engineered *E. coli* strains managed to degrade the uric acid (250 µM) found in the media completely in 24 h. Possible effect of media composition on gene expression should be taken into consideration while comparing the uric acid degradation capacity of each genetic construct (pBR-pucLM or pAC-ygfU). M9MM is formulated to optimize bacterial growth under stringent conditions. Whereas, FaSSIF-V2 media is formulated to simulate human intestinal fluid, which might not be supportive of maximal recombinant gene expression.

Finally, we designed an *in vitro* Caco-2 model to study the uric acid transport and degradation in an environment that mimics the human intestinal lumen. Our FACS analysis results (Supplementary Figure S3) and significant reduction in uric acid transport in the presence of elacridar, the BCRP inhibitor, indicated the abundant expression levels of BCRP transporter on Caco-2 cells (Figure 7B). As for the uric acid degradation studies on Caco-2 monolayer, we worked with pBR-pucLM and pAC-ygfU harboring *E. coli* K-12 MG1655 because MG1655 chassis gave promising results as a uric acid bioreporter, as well. As seen in Figure 7C, engineered bacteria significantly reduced the apical uric acid concentrations. However, the presence of native MG1655 in the apical compartment did not cause a change. Further, the temporal dynamics of uric acid pumping and degradation can be compared to the dynamics of only pumping or only degrading uric acid in Figure 7C. Strains without both capabilities removed uric acid comparably to the strain with both in the first 2 h. After 2 h, the amount of uric acid in the apical space increases so that by 6 h, only the strain with both capabilities was able to significantly reduce uric acid levels. We showed that the Caco-2 *in vitro* model is an efficient test bed to investigate the potential of engineered *E. coli* K-12 MG1655 for elimination of excess uric acid through the human intestinal tract.

The safe utilization history of K-12 strains (US EPA, 1997) implies that *E. coli* K-12 MG1655 would a good candidate for human use. K-12 strains cannot colonize the human intestinal tract under normal conditions due to their inability to bind the mucosal surface of the colon. NIH guidelines classifies K-12 strains under Class 1, minimal or no hazard category (Department of EHS, 2017). Moreover, the United States Environmental Protection Agency (US EPA) reports that these strains are very unlikely to pose a risk for the environment or human health (US EPA, 1997). Evidence from previous studies demonstrated that *E. coli* K-12 strains can survive in the human intestine up to 6 d, without colonization or transfer of plasmid DNAs to endogenous gut bacteria (Levy et al., 1980). This time period might be sufficient to achieve accurate hyperuricemia diagnosis and significant reduction in serum uric acid levels. For clinical diagnosis, the bioreporter module presented in this study can be modified to control production of a dye, such as indigo (Bhushan et al., 2000), which in turn can be detected and quantified in patient stool samples. Once hyperuricemia diagnosis is achieved, a treatment regimen can be implemented using *E. coli* K-12 equipped with uric acid degradation module (i.e., overexpression of native *E. coli* uric acid transporter, YgfU and recombinant *B. subtilis* urate oxidase, PucLM). A recent study by Zhao and co-workers (Zhao et al., 2022) demonstrated a significant reduction in mouse uric acid levels following intravenous injection of uric acid. While the method of generating hyperuricemia was unorthodox (Zhao et al., 2022), the result was supportive of our findings. Further, in that study an oxidation module was added to the cassette for increasing the rate of uric acid degradation. While we found this compelling, there is also some risk associated with too rapid clearing of uric acid. Uric acid is a strong antioxidant, and it may require a longer presence in the GI tract than 1 h. Further studies on animal models will be invaluable to determine the dosage of bacteria (CFU/g) and optimal intervals of administration required for these desired outcomes.

In conclusion, in this study, we presented *E. coli* equipped with synthetic gene networks, as a potential novel cell-based synthetic therapeutic for detection of hyperuricemia and degradation of uric acid. Our findings indicate that engineered commensal *E. coli* K-12 MG1655 has the potential to be used both for uric acid detection and degradation in humans.

## Materials and methods

### Strains and growth conditions

Bacterial strains used in this study are listed in Supplementary Table S1. *E. coli* DH5α, *E. coli* K-12 MG1655, and *E. coli* Nissle 1917 were used as hosts for plasmid construction and recombinant protein expression. For chromosomal isolation of *pucL*, and *pucR* genes, *B. subtilis* 168, and for the isolation of *ygfU* gene, *E. coli* K-12 MG1655 was used. All strains were maintained in Luria-Bertani (LB) broth at 37°C with 250 rpm shaking for routine growth. For GFP assays, M9 minimal medium (M9MM) (Cold Spring Harb. Protocols, 2010) and Fasted State Simulated Intestinal Fluid (FaSSIF-V2) (Jantratid et al., 2008) supplemented with 0.4% glucose was prepared. For *E. coli* DH5α cultures during GFP assay, 1 μg/mL thiamine was added into M9MM and FaSSIF-V2. For all experiments, each media type was supplemented with appropriate antibiotics: ampicillin 100 μg/mL, chloramphenicol 25 μg/mL, tetracycline 10 μg/mL, and kanamycin 25 μg/mL.

### Plasmid construction

Plasmids and primers used in this study are listed in Supplementary Tables S2, S3. Synthetic promoter sequence, *pucpro*, ordered as 149 bp long, 4 nmole Ultramer^®^ DNA Oligo from Integrated DNA Technologies (IDT). Q5^®^ High-Fidelity DNA Polymerase (NEB^®^) was used in all PCR reactions. PCR amplified plasmid backbones and inserts were assembled using Gibson Assembly^®^ Master Mix (NEB^®^). All plasmid sequences were verified via DNA Sanger Sequencing (Cornell Biotechnology Resource Center, Genomics Facility).

### Uric acid stock

An initial stock of uric acid (50 mM) was prepared by dissolving uric acid (Sigma-Aldrich^®^) in 400 mM NaOH. If necessary, this stock solution was diluted in sterile dH_2_O to get desired uric acid concentrations in growth media.

### GFP assay

To measure bioreporter’s performance, fluorescent assays were conducted using Synergy 4 Plate Reader, BioTek^®^ (Winooski, VT). 485/20 excitation filter and 528/20 emission filter were used to record relative fluorescence unit (RFU) of each sample. *E. coli* DH5α carrying pGFPuv plasmid was used as a positive control to ensure GFP assay and plate reader settings were working fine. To induce GFP gene expression from pGFPuv, media was supplemented with 0.2 mM Isopropyl β-D-1-thiogalactopyranoside (IPTG) (VWR™). Single colonies of *E. coli* DH5α, K-12 MG1655, Nissle 1917, and engineered *E. coli* containing bioreporter constructs were inoculated into LB. After 18 h of incubation, cultures were diluted 1:25 in M9MM or FaSSIF-V2 supplemented with different uric acid concentrations: 0 μM, 50 μM, 62.5 µM, 75 μM, 87.5 µM, 100 μM, 150 μM, and 250 µM. Diluted cultures were grown at 37°C shaker for at least 6 h. Finally, RFU was measured for each sample and fluorescence was normalized to cell density (OD600) during data analysis.

### Colorimetric uric acid assay

Single colonies of *E. coli* DH5α, K-12 MG1655, Nissle 1917, and engineered *E. coli* strains harboring uric acid degradation constructs were inoculated into LB. After 18 h of incubation, cultures were diluted 1:25 in M9MM or FaSSIF-V2 supplemented with 250 µM uric acid. These diluted cultures were grown in M9MM or FaSSIF-V2 for 24 h at 37°C. Overnight cultures were spun down at 3,000 rpm for 10 min. Supernatant was collected to measure remaining uric acid concentration in the media using colorimetric uric acid assay kit (Eton Bioscience Inc) against a uric acid standard curve of known concentrations.

### Uric acid transport and degradation using *in vitro* Caco-2 model

Caco-2 cells were obtained from ATCC^®^ (HTB-37™). Cells were cultured in Dulbecco’s Modified Eagle Medium - DMEM - supplemented with 10% fetal bovine serum albumin - FBS - and 1 × antibiotic-antimycotic solution (Gibco™) at 37°C under an atmosphere of 5% CO_2_ in the air. For uric acid transport and degradation studies, Caco-2 cells were seeded on 12mm, 0.4 μm Transwell^®^ inserts (Corning^®^). Seeded cells were cultured for 16–18 d, and Caco-2 monolayer integrity was assessed via Lucifer Yellow % passage assay before each experiment (HTS_Transwell_96_Protocols_Drug_Transport_CLS, 2019). Monolayers with <3% LY passage were used in the following setups.

For the uric acid transport experiments, uric acid supplemented transport medium–1 × Hank’s Balanced Salt Solution, 10 mM HEPES pH: 7.4, 25 mM glucose–was added to the basolateral compartment and transport medium without uric acid was added to the apical compartment of Transwell^®^ inserts. For the treatment group, 25 μM elacridar (Sigma-Aldrich^®^)–BCRP inhibitor–was dissolved in DMSO and added to the apical side. For the control group, only DMSO was added to the apical side. Monolayers were incubated at 37°C, 5% CO_2_ for up to 8 h, and aliquots were collected at every 2 h from apical compartment to measure the change in uric acid concentrations.

For the uric acid degradation studies on Caco-2 monolayers, 2.5 mM uric acid supplemented transport medium was added to the basolateral compartment and transport medium without uric acid was added to the apical compartment. These Transwell^®^ setups were incubated at 37°C, 5% CO_2_ to enable uric acid transport into the apical chamber. As for the bacteria, wild-type *E. coli* K-12 MG1655 and engineered strains with uric acid degradation module were grown in LB medium at 37°C for 12–16 h. Overnight cultures were spun down at 3,000 rpm for 10 min, and supernatant was removed. Cell pellets was resuspended in DMEM media without phenol red (Gibco™) and with FBS (Biowest), and diluted to OD600 = 1.0. The diluted culture was added into the apical chamber of previously prepared Transwell^®^ setups, giving the final OD600 of 0.1. These bacteria supplemented setups were incubated at 37°C, 5% CO_2_ for up to 6 h and aliquots (100 μL) were collected from apical compartment to measure the change in uric acid concentrations.

### FACS analysis

Caco-2 cells were seeded on 12mm, 0.4 μm Transwell^®^ inserts (Corning^®^) at a density of $5*10^{4}\frac{cells}{{cm}^{2}}$ , and incubated at 37°C, 5% CO_2_ for 21 d. During this period, cells were cultured in 10% FBS and 1 × antimycotic-antibiotic supplemented DMEM, which was replaced with fresh media every 2–3 d. After 21 d, Caco-2 monolayer was washed with 1 × PBS, and cells were treated with trypsin-EDTA 0.25% solution (Gibco™) for 20 min, and centrifuged at 300 RCF for 5 min. Harvested cells were washed in ice cold FACS buffer (1 × PBS, 0.5% BSA, 0.1% NaN_3_), and their numbers were adjusted to $1*10^{6}\frac{cells}{ml}$ . Next, Caco-2 cells were labeled with a PE-conjugated BCRP (ABCG2) monoclonal antibody (Invitrogen, Catalog # 12-8888-42) following a FACS cell surface staining protocol (M edicine.yale, 2020).

All FACS analysis were performed using the Thermo Fisher Attune™ NxT Flow Cytometer (Cornell Biotechnology Resource Center, Flow Cytometry Facility).

### Statistical analysis

All statistical analysis was made in GraphPad Prism 7.04 software. Results were presented as mean ± s.d., unless stated otherwise. To calculate the goodness of fit (R^2^), linear regression analysis was performed. Student’s t-test (*α* = 0.05) was conducted to compare apical uric acid concentrations under two different conditions during *in vitro* Caco-2 experiments (Figures 7B, C). For statistical analysis of multiple group comparisons, one-way ANOVA (*α* = 0.05) was combined with Bonferroni’s correction.

## Data Availability

The original contributions presented in the study are included in the article/Supplementary Material, further inquiries can be directed to the corresponding author.
