# Supplementary material for: Engineering Escherichia coli for diagnosis and management of hyperuricemia
Source: Front Bioeng Biotechnol. 2023 May 23;11:1191162. doi: 10.3389/fbioe.2023.1191162 (PMC10242094; doi:10.3389/fbioe.2023.1191162)
Supplement: Supplementary file 1 [file DataSheet1.docx]

**SUPPLEMENTARY INFORMATION**


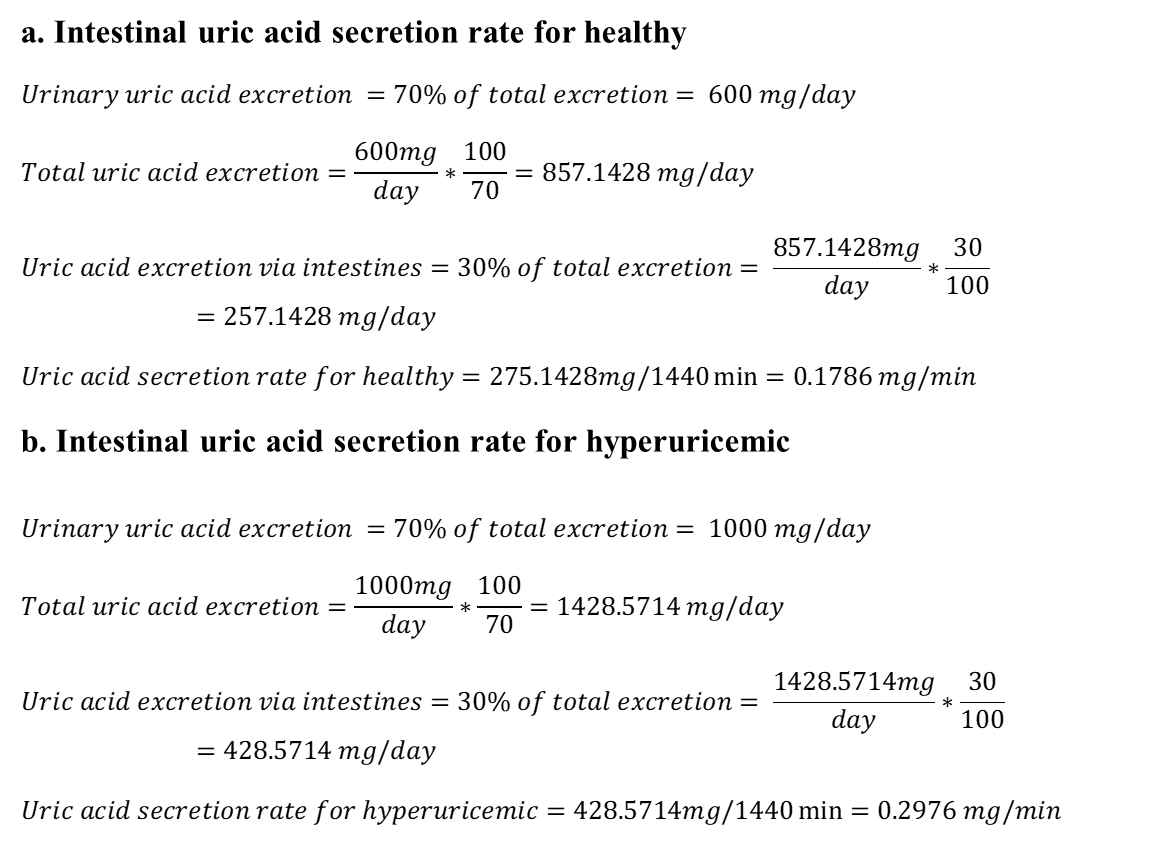


**Supplementary Figure 1.** Calculation of intestinal uric acid secretion rates for **(a)** healthy individuals and **(b)** hyperuricemia patients.

**
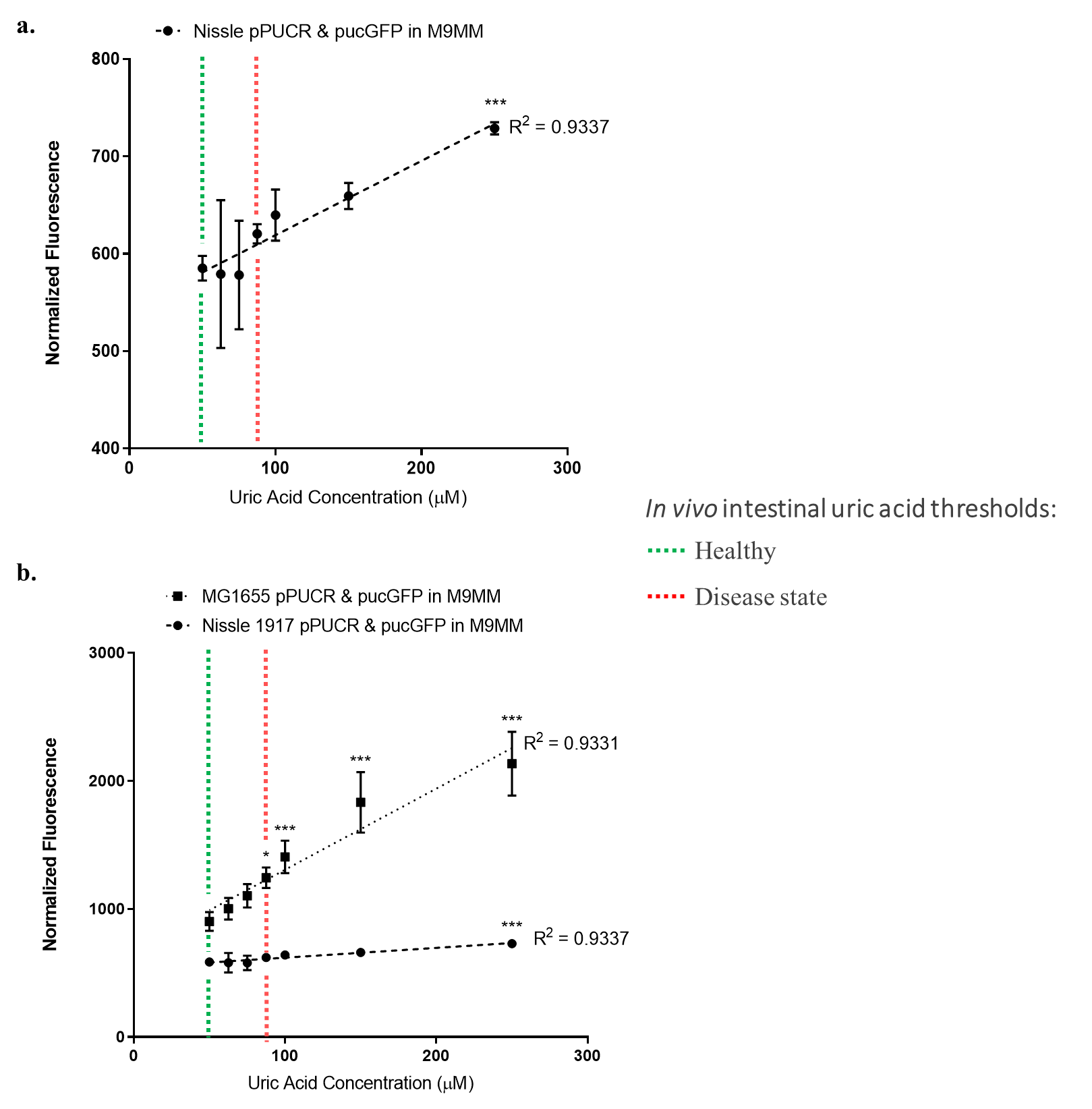
**

**Supplementary Figure 2.** Characterization of uric acid bioreporter in probiotic *E. coli* Nissle 1917 in response to increasing uric acid concentrations: 50μM, 62.5μM, 75μM, 87.5μM, 100μM, 150μM, and 250μM in M9MM. **(b)** Comparison of bioreporter module’s performance in Nissle 1917 to K-12 MG1655. Each data point shows the mean normalized fluorescence and s.d. (error bars) obtained from 4 biological replicates. The mean of each data point was compared to the mean of the control group, 50µM uric acid. * and *** indicates statistical significance at *P* < 0.05 and *P* < 0.001 respectively. Statistical analysis: one-way ANOVA is combined with the Bonferroni multiple comparisons test $(a=0.05)$.

**
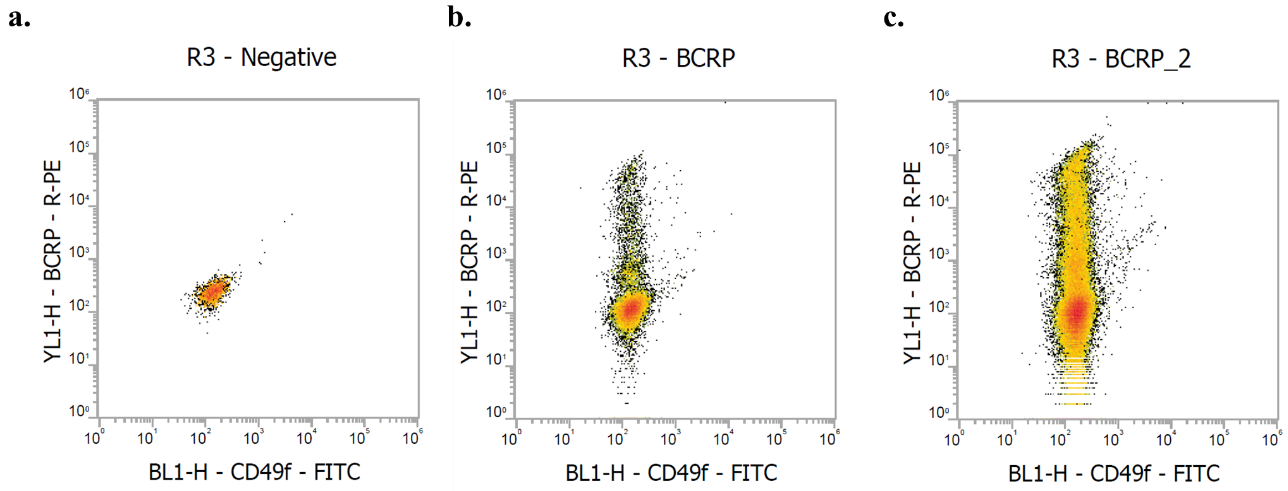
**

**Supplementary Figure 3.** FACS analysis results for BCRP expression in Caco-2, human intestinal epithelial cell line. Analyzed samples include **(a)** unstained Caco-2 cells as the negative control, and **(b & c)** replicates of Caco-2 cells that are stained with PE-conjugated anti-BCRP antibody.

**Supplementary Figure 4.** qPCR results for BCRP expression. *** indicates statistical significance at 𝑝 < 0.001. Statistical analysis: unpaired student’s t-test $(a=0.05)$. Caco-2 BCRP expression level is significantly lower compared to wild type small intestine cells. Based on these findings, basolateral compartment of *in vitro* Caco-2 model was supplemented with 2.5 mM uric acid, concentrations higher than serum levels seen in patients. This enabled the *in vitro* model to better mimic luminal uric acid concentrations where engineered *E. coli* strains’ ability to remove excreted uric acid was assessed.

| *Supplementary Table 1* | | |
| --- | --- | --- |
| Strain Name | **Genotype / Description** | **Source** |
| *E. coli* DH5α | *fhuA2 Δ(argF-lacZ)U169 phoA glnV44 Φ80 Δ(lacZ)M15 gyrA96 recA1 relA1 endA1 thi-1 hsdR17* | March Group |
| *E. coli* K-12 MG1655 | F^–^ λ^–^ rph-1 | March Group |
| *E. coli* Nissle  1917 | Wild type *Escherichia coli* strain with probiotic properties | March Group |
| *B. subtilis* 168 | *trpC2* | Helmann Group |
| JW5470-1 | F- Δ(araD-araB)567, ΔlacZ4787(::rrnB-3), λ-, **ΔygfU738::kan**, rph-1, Δ(rhaD-rhaB)568, hsdR514 | CGSC |

| *Supplementary Table 2* | | |
| --- | --- | --- |
| Plasmid Name | **Description** | **Source** |
| pACYC184 | p15A origin of replication  **Antibiotic markers:** CAT and TetR  **Promoters:**   - TetR promoter P1: AGTTTATCACAGTTAAATTGCTAACGCAGTCA - TetR promoter P2: TTGACAGCTTATCATCGATAAGCTTTAAT - CAT promoter: ACGTAAGAGGTTCCAACTTTCACCATAAT | March Group |
| pGFPuv | pBR322 origin of replication, GFPuv gene under the control of lac promoter  **Antibiotic marker:** AmpR  **Promoters:**   - Lac promoter: TTTACACTTTATGCTTCCGGCTCGTATGTT - AmpR promoter: TTCAAATATGTATCCGCTCATGAGACAAT | March Group |
| pBR322 | pMB1 origin of replication  **Antibiotic markers:** AmpR and TetR  **Promoters:**   - TetR promoter P1: AGTTTATCACAGTTAAATTGCTAACGCAGTCA - TetR promoter P2: TTGACAGCTTATCATCGATAAGCTTTAAT - AmpR promoter: TTCAAATATGTATCCGCTCATGAGACAAT | March Group |
| pPUCR | TetR marker and promoter removed from pACYC184 backbone and replaced with  *B. subtilis* 168 *pucR* promoter and gene.  **Antibiotic marker:** CAT | This work |
| pucGFP | lac promoter from pGFPuv backbone removed and replaced with pucpro synthetic promoter.  **Antibiotic marker:** AmpR | This work |
| pBR-pucLM | AmpR marker removed from pBR322 backbone and *pucLM* gene fusion from *B.* *subtilis* cloned under AmpR promoter’s control.  **Antibiotic marker:** TetR | This work |
| pAC-ygfU | TetR marker removed from pACYC184 backbone and ygfU gene from *E. coli* K12 MG1655 cloned under TetR promoter (P2).  **Antibiotic marker:** CAT | This work |

| *Supplementary Table 3* | | |
| --- | --- | --- |
| 1. *Primers used for bioreporter module assembly* | | |
| Primer Name | **Sequence** | **Source** |
| pucpro promoter | GTTGGCCGATTCATTAATGCGGCACGTAAGATATCATTGGTTAAACCATAATGAAATAAGATCACTACCGGGCGTATTTTTTGAGTTATCGAGATTTTCAGGAGCTAAGGAAGCTAAAATGAGTAAAGGAGAAGAACTTTTCACTGGAG | This work |
| Amplification of pGFPuv backbone | | |
| pGFPuv lin F | GGTGCCTCACTGATTAAGCATTGGTAACTGTCAGACCAAGTTTACTC | This work |
| pGFPuv lin R | GAGTAAACTTGGTCTGACAGTTACCAATGCTTAATCAGTGAGGCACC | This work |
| Amplification of pucR from *B. subtilis* 168 genomic DNA | | |
| pucR ins F | GTAGCACCTGAAGTCAGCCCCATGTCAGTTTATGTAACACAACCAG | This work |
| pucR ins R | GAGGCAGACAAGGTATAGGGCGGTGTCATTGGATACAGTAGCTG | This work |
| Amplification of pACYC184 backbone | | |
| pucR-184 lin F | GCCCTATACCTTGTCTGCCTC | This work |
| pucR-184 lin R | GGCTGACTTCAGGTGCTACATTTG | This work |
| 1. *Primers used for degradation module assembly* | | |
| Amplification of ygfU gene from *E. coli* K-12 MG1655 genomic DNA | | |
| proTet-ygfU-GA-Fwd | GCAGTCAGGCACCGTGTATGAGCGCCATAGATTC | This work |
| ACYC184-ygfU-GA-Rev | GGAGTGGTGAATCCGTTTATTCTCCATGCTCATTTTTC | This work |
| Amplification of pACYC184 backbone | | |
| pTetR-GacA-Rev | ACACGGTGCCTGACTGCGTTAG | March Group |
| pBR-GacA-lin-Fwd | ACGGATTCACCACTCCAAGAATTGG | March Group |
| Amplification of pucLM fusion from *B. subtilis* 168 genomic DNA | | |
| pBR-pucM-GA-Rev | GAGTAAACTTGGTCTGACAGTTAACTCCCCCTATAC | This work |
| AmpRPro-pucL-GA-Fwd | TATTGAAAAAGGAAGAGTATGTTCACAATGGATGACCTGAACC | This work |
| Amplification of pBR322 backbone | | |
| pAC177-lin-Fwd-01/16 | CTGTCAGACCAAGTTTACTCATATATACTTTAG | This work |
| Pro-bla-Rev | ACTCTTCCTTTTTCAATATTATTGAAGCATTTATCAG | This work |
